# Different Causes of Functional Tricuspid Valve Regurgitation Are Linked to Differences in Tricuspid Valve and Right-Sided Heart Geometry and Function: 3D Echocardiography Study

**DOI:** 10.3390/medicina59010057

**Published:** 2022-12-27

**Authors:** Aušra Krivickienė, Dovydas Verikas, Rita Krečkauskienė, Lina Padervinskienė, Deimantė Hoppenot, Skaidrius Miliauskas, Justina Jolanta Vaškelytė, Eglė Ereminienė

**Affiliations:** 1 Department of Cardiology, Medical Academy, Lithuanian University of Health Sciences, LT-44307 Kaunas, Lithuania; 2 Institute of Cardiology, Lithuanian University of Health Sciences, LT-50162 Kaunas, Lithuania; 3 Department of Radiology, Medical Academy, Lithuanian University of Health Sciences, LT-44307 Kaunas, Lithuania; 4 Department of Pulmonology, Medical Academy, Lithuanian University of Health Sciences, LT-44307 Kaunas, Lithuania

**Keywords:** tricuspid valve, tricuspid regurgitation, 3D-echocardiography

## Abstract

*Background and Objectives:* The aim of this study was to clarify the tricuspid valve (TV) and right ventricular (RV) geometry and function characteristics using 3D echocardiography-based analysis and to identify echocardiographic predictors for severe tricuspid regurgitation (TR) in different etiologies of functional TR (fTR). *Methods and Results*: The prospective study included 128 patients (median age 64 years, 57% females): 109 patients with moderate or severe fTR (69-caused by dominant left-sided valvular pathology (LSVP), 40 due to precapillary pulmonary hypertension (PH)), and 19 healthy controls. The 2D and 3D-transthoracic echocardiography analysis included TV, right atrium, RV geometry, and functional parameters. All the RV geometry parameters as well as 3D TV parameters were increased in both fTR groups when compared to controls. Higher RV diameters, length, areas, volumes, and more impaired RV function were in PH group compared to LSVP group. PH was associated with larger leaflet tenting height, volume, and more increased indices of septal-lateral and major axis tricuspid annulus (TA) diameters. LVSP etiology was associated with higher anterior-posterior TA diameter and sphericity index. Univariate and multivariate logistic regression and ROC analyses revealed that different fTR etiologies were associated with various 2D and 3D echocardiographic parameters to predict severe TR: major axis TA diameter and TA perimeter, the leaflet tenting volume had the highest predictive value in PH group, septal-lateral systolic TA diameter-in LSVP group. The 3D TA analysis provided more reliable prediction for severe fTR. *Conclusions*: TV and RV geometry vary in different etiologies of functional TR. Precapillary PH is related to more severe RV remodeling and dysfunction and changes of TV geometry, when compared to LSVP group. The 3D echocardiography helps to determine echocardiographic predictors of severe TR in different fTR etiologies.

## 1. Introduction

The most common cause of tricuspid regurgitation (TR) is not a primary TV disease (organic TR) but rather impaired valve coaptation (secondary or functional TR) caused by dilatation of the right ventricle (RV) and/or of the tricuspid annulus (TA) due to left-sided heart valve diseases, pulmonary hypertension, congenital heart defects, atrial fibrillation, and cardiomyopathy [1,2]. 

Functional TR (fTR) and left-sided cardiac or pulmonary diseases have been linked for a long time. Given this, the link between fTR and excessive afterload in pulmonary hypertension is seen as the core fTR mechanism and is the focus of guidelines for valve diseases [3,4]. However, fTR remains a frustrating and poorly understood condition that is associated with decreased survival, morbidity, and functional capacity [5,6,7]. With the recognition of the disease’s progressive nature and the impact of secondary TR on outcomes in a variety of patient conditions [5,6,7,8,9], there has been an increase in interest in understanding the pathophysiology and mechanisms of fTR and its association with RV remodeling.

Echocardiography is the first-line imaging modality for assessing TV geometry, the presence, severity, and mechanism of TR, as well as its effects on the RV. Traditional 2D echocardiography is unsuitable for studying the anatomy and pathophysiologic mechanisms of the regurgitant TV due to its complex three-dimensional (3D) geometry and anterior position in the mediastinum. The 3D echo has become an integral and essential tool for assessing TV morphology, defining the mechanism of TR, and assessing volumes and function of the right atrium and ventricle [10,11,12,13]. There is currently a scarcity of precise 3-dimensional echo-based research into how the various etiologies of fTR affects right-sided heart geometrical and functional deformation.

The aim of this study was to clarify the TV and RV geometry and function characteristics using 3D echocardiography-based analysis and to identify echocardiographic predictors for severe TR in different etiologies of functional TR.

## 2. Materials and Methods 

### 2.1. Study Population 

In the prospective study, which took place from July 2018 to December 2021, 2D and 3D echocardiographic evaluations were done on 128 patients, 109 of whom had functional moderate or severe TR and 19 of whom were healthy controls. FTR patients were divided into two groups according to the different etiologies of fTR: 1. fTR caused by dominant left-sided valvular pathology (LSVP)–69 patients; 2. fTR caused by precapillary pulmonary hypertension (PH) (invasively measured pulmonary capillary wedge pressure < 15 mmHg)–40 pts. Patients with ischemic heart disease (assessed by coronary angiography), chronic pulmonary disease or congenital heart disease were excluded from the study. Additionally, measurements of 3D TV and right heart parameters were obtained from 19 healthy volunteers who have been sent to the echocardiography laboratory as controls. TR or heart failure was ruled out by a physical examination, and echocardiography. No controls had any echocardiographic abnormality including TV or right-sided chambers and thus could be considered as having normal TV geometry. 

The study was approved by Kaunas Regional Biomedical Research Ethics Committee, No. BE-2–64, issued on 24 July 2018. All patients provided informed consent.

### 2.2. Transthoracic Echocardiography

The 2-dimensional and 3-dimensional echocardiography was performed using a GE VingMed VividE95 (GE Vingmed Ultrasound AS, Horten, Norway) imaging system, equipped with a M3S 4.0 MHz transducer, capable of displaying 3D images. An experienced independent echocardiographer blinded to the patient’s clinical data performed the echocardiographic studies. Digital loops were stored and analyzed offline (GE Vingmed, Phillips TomTec, Unterschleissheim, Germany). The 3D TV analysis was made using 4D Auto TVQ quantification software package (GE Healthcare, Horten, Norway).

**Figure 1:**
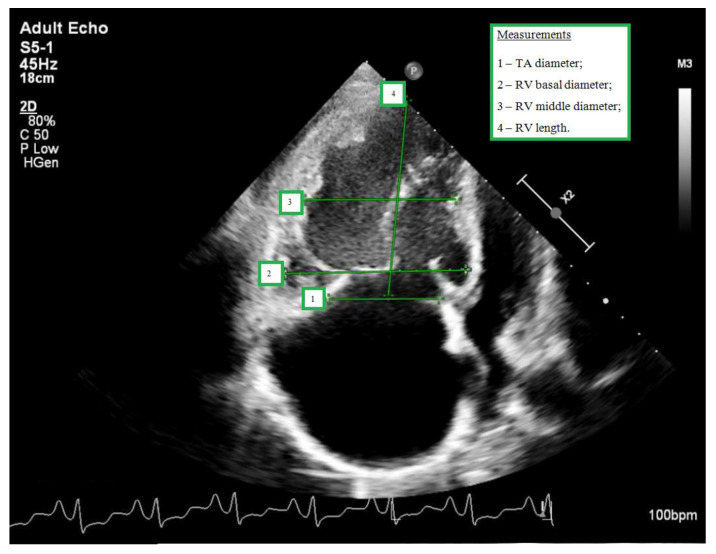
The 2D RV and TV echocardiographic measurements.

Anatomic and Doppler examinations and measurements were performed according to recent American Society of Echocardiography recommendations and European Association of Cardiovascular Imaging (EACVI) guidelines [2,14]. The 2D and 3D echocardiography was performed and included the following parameters: the left ventricular (LV) and left atrial geometry (diameters and volumes), LV ejection fraction, the RV geometry (RV diameters, areas and volumes), functional (velocity of the tricuspid annular systolic motion (S’), tricuspid annulus plane systolic excursion (TAPSE), RV fractional area change (FAC), RV ejection fraction (RVEF), and strain (RV free wall longitudinal strain (RV FWLS) and RV septal longitudinal strain (RV SLS)), the tricuspid valve 2D (the systolic and diastolic 4-chambers, leaflet tenting height and area), and 3D (the systolic and diastolic 4-chambers (septal–lateral), 2-chambers (anterior-posterior), major and minor axis TA diameters, TA area, perimeter, leaflet tenting height and volume), right atrium (RA) (diameter, length, area and volume) parameters (Figure 1). Parameters have been indexed to body surface area.

Volume datasets were obtained under breath-hold to avoid stitch artifacts using multi-beat full volume model in the four-chamber apical view focused on the RV and using multi-beat 3D zoom mode in the four-chamber apical view focused on the TV. The views were optimized for depth and gain setting before 3D acquisition and close attention was given to including the entire TV or RV in the sector boundaries. A multislice display was used during acquisition to ensure a complete inclusion of the RV in the dataset [15]. The RV and TV multi-beat 3D views dataset was also acquired with the narrowest possible depth under breath-hold to obtain a higher volume rate (higher than 20 volumes per second). 

**Figure 2:**
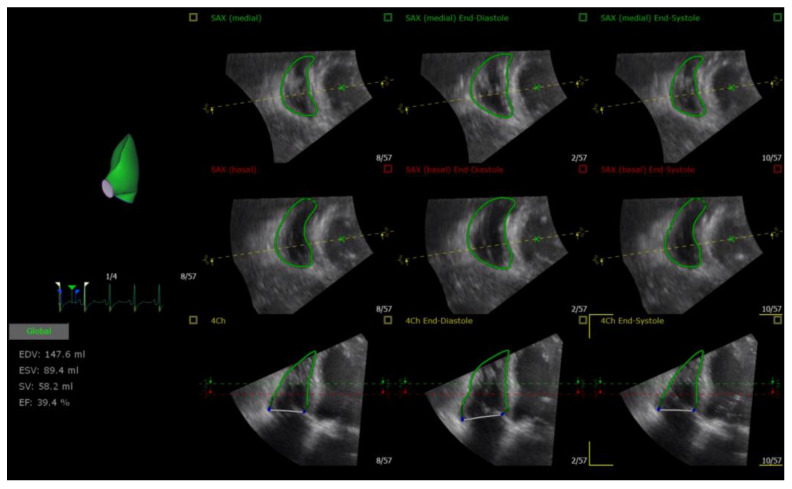
The 3D RV echocardiographic measurements.

The digitally stored multi-beat full volume dataset in the apical axis four-chamber view was imported into the dedicated software tools (4D RV- Analysis, TomTec Imaging Systems, Unterschleissheim, Germany) to calculate RV end-diastolic and end-systolic volumes and RVEF (Figure 2).

**Figure 3:**
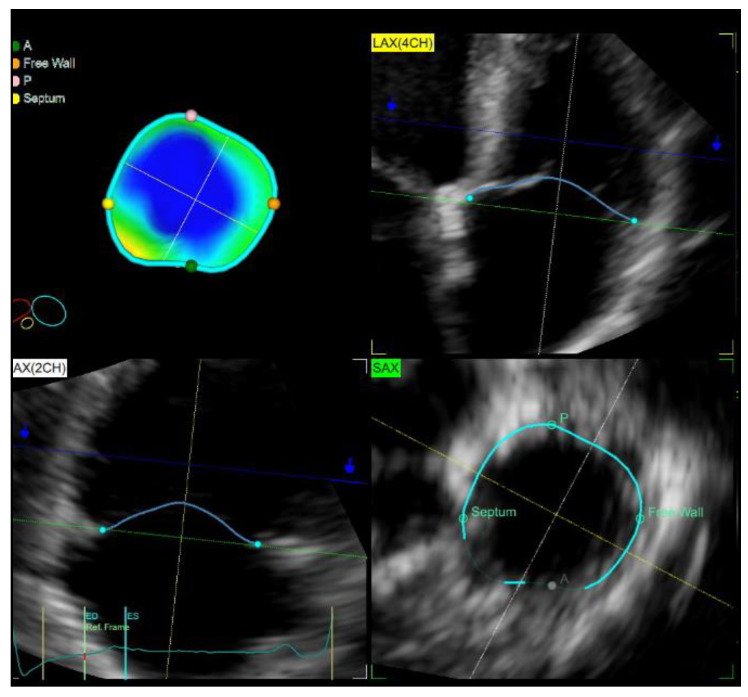
The 3D TV echocardiographic measurements.

The digitally stored live 3D zoom dataset was imported into the 4D Auto TVQ quantification (GE Healthcare, Horten, Norway) workstation to analyze the TV geometry (Figure 3). Anterior–posterior and septal–lateral diameters of TV as well as major and minor axis diameter, the TV annular area, perimeter, and sphericity index were obtained on a midsystolic and middiastolic frame. The closed leaflets were traced in midsystole on successive equidistant long-axis planes to obtain the leaflet tethering height and 3D tenting volume. 

Continuous wave Doppler was used to assess maximal tricuspid regurgitation flow velocity (V) to estimate the systolic pressure gradient (4V^2^) between the RV and RA. Pulmonary artery systolic pressure was calculated by adding an estimated RA pressure. The mean pulmonary artery pressure was calculated using 80–(PA acceleration time/2) formula. The severity of TR was measured quantitatively according to the recent ESC guidelines [4], using TR effective regurgitant orifice area (EROA) (PISA) and biplane TR vena contracta (VC) (the width of the color jet at its narrowest point) from 2D apical four chamber view. 

According to a recently proposed grading scheme for TR, patients with biplane VC 3–6.9 mm and TR EROA 20–39 mm^2^ were considered as moderate and VC ≥ 7 mm and EROA ≥ 40 mm^2^ were considered as severe TR [4,16,17].

### 2.3. Statistical Analysis

Continuous variables are expressed as median (interquartile range (IQR)) and were compared by non-parametric Wilcoxon rank-sum tests. Categorical variables are presented as number (percentage). All variables were tested for normality using the Shapiro–Wilk test. Univariable and multivariable logistic regression analyses were performed to identify right heart and TV parameters associated with severe fTR. Variables that demonstrated a significant association with the outcome in the univariate analysis were then included in the multivariable-adjusted analysis to describe how the factors jointly predict severity of TR. Results were expressed as odds ratio (OR), with 95% confidence intervals (95% CI). The optimal model selection involved finding the best compromise between the number of factors to keep and the accuracy of the logistic regression analysis. Receiver-operating characteristic (ROC) analysis was conducted to determine the optimal cut-off value of the 2D and 3D echocardiographic parameters for predicting severe fTR. The accuracy of this cut-off ratio was evaluated using the area under the ROC curve (AUC). Statistical analyses of the data were performed using SPSS version 27 (IBM, Armonk, NY, USA). *p*-values of <0.05 were considered statistically significant. 

## 3. Results

### 3.1. Study Population 

Clinical characteristics of the study population are detailed in Table 1. Patients in the PH group were younger and predominantly female. The body mass index did not vary between any of the three groups.

### 3.2. Comparison and Difference in 3D TV Geometry and Right Heart Remodelling between Controls and fTR

Table 1 and Table 2 display the echocardiographic characteristics of patients from various fTR etiologies. The severity of TR did not differ between both fTR groups. All the RV geometry parameters as well as 3D TV parameters were increased in fTR groups when compared to controls. Larger RV end-diastolic volume and area were found in the LSVP group when compared to normal controls (*p* < 0.001; *p* < 0.001, respectively). LSVP patients experienced a 104% increase in RA volume (*p* < 0.001). 

In comparison to normal controls, PH patients showed a 63% increase in the TV annular area in midsystole, a 31% increase in septal-lateral, 28% in major TA diameter, and a 222% increase in tenting volume (all *p* < 0.01; Table 2).

### 3.3. Comparison of Left and Right Heart Remodelling and 3D TV Geometry between Different Etiology fTR Groups

The LV ejection fraction did not differ between the fTR groups LSVP and PH groups (52 vs. 51%; respectively, *p* = 0.928), despite the fact that the diameters and volumes of the LV and the left atrium were substantially enlarged in the LSVP group compared with PH (LV end-diastolic diameter index: 27.9 vs. 22.8 mm/m^2^; respectively, *p* < 0.001; LA volume index: 58.8 vs. 27.0 mL/m^2^; respectively, *p* < 0.001).

Even though the RA area (RAA) and TR severity, measured quantitatively, did not differ between the fTR groups (*p* = 0.484 for RAA and *p* = 0.464 for TR EROA), the differences of TV and RV geometry and functional parameters were found.

### 3.4. Comparison of RV Geometry and Function between Different Etiology fTR Groups

PH was associated with larger RV basal and middle diameters, and larger RV end-diastolic and end-systolic area and volume, compared to LSVP group. More impaired RV functional parameters (S’, FAC, RVEF and RV lateral wall strain) and higher systolic and mean pulmonary artery pressures were also found in precapillary PH group (Table 1 and Table 2).

### 3.5. Comparison of TV Geometry between Different Etiology fTR Groups

Although the 2D measured TA systolic and diastolic diameters did not differ between fTR groups, the 3D-echo TA analysis revealed additional and more precise information. Precapillary PH was associated with more increased indices of TA systolic and diastolic septal-lateral and major axis diastolic diameters. Meanwhile, LVSP etiology was associated with larger anterior-posterior diameter and higher TA sphericity index compared to PH group. Larger leaflet tethering, tenting area, and volume were found in PH group.

### 3.6. Relation of 3D TV and RV Geometry and Severity of fTR

To assess the impact of TR severity on the TV geometry and right heart remodeling, regression analysis was performed. Analysis included 34 pts (31.5%) with severe fTR and 74 pts (68.5%) with moderate fTR. To define which parameters had highest predictive value in all cohort, ROC analysis was performed (Supplement Table S1). Parameters with AUC > 0.7 were included in further analysis. Univariable and multivariable analyses are shown in Table 3. Both area and length parameters of TV were shown to have predictive values for identification of fTR severity. The changes of 3D echocardiographic TA diameters had better prediction of fTR severity than 2D echo measured TA diameters (3D septal-lateral systolic diameter OR 1.507 vs. 2D 4-chambers systolic diameter OR 1.255 (Table 3)). However, only 3D measured septal-lateral systolic diameter and TA perimeter were independent predictors of TR severity when analysis was performed together with right heart parameters. 

### 3.7. Prediction of Severe fTR in Different fTR Etiologies

**Figure 4:**
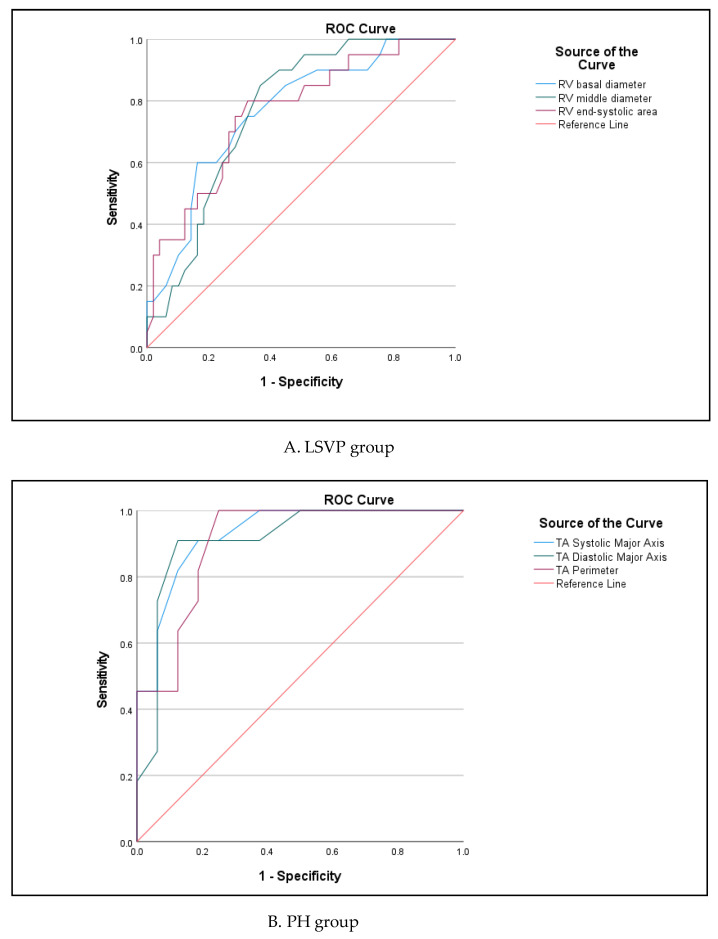
ROC analysis for prediction of severe fTR.

ROC analysis of selected echocardiographic parameters with highest predictive value in different etiology is presented in Supplement Table S2 and Figure 4. Distribution of severe fTR did not differ between the groups (29% in LSVP vs. 35% in PH, *p* > 0.05). RV parameters analysis demonstrated that RV middle diameter (AUC 0.790) and RVEF (AUC 0.786) had the best predictive value for severe fTR in PH group, while, of all TV parameters, systolic (AUC 0.929) and diastolic (AUC 0.912) major axis, and annulus perimeter (AUC 0.906) had the highest predictive value in this group of pts. The 3D TA analysis provided more reliable prediction for defining PH pts with severe fTR than 2D echocardiography (sensitivity 82–91% for 3D vs. 64–71% for 2D). The leaflet tenting volume had the predictive value for severe fTR in PH, but not in LSVP group. 

Remarkably, of 2D parameters, RV middle (AUC 0.768) and basal (AUC 0.765) diameter, together with RV end-systolic area (AUC 0.764) were related to severe fTR in LSVP group. Meanwhile, RVEF lost the significance in predicting severe fTR in this group of pts. ROC analysis revealed that 3D echocardiography derived TV parameters still had predictive value for severe fTR in LSVP group, with the highest predictive value (AUC 0.761) for septal-lateral systolic TA diameter. 

Those parameters which showed higher sensitivity and specificity were included in univariate logistic regression (Table 4). In the PH group, diameter (septal–lateral, anterior–posterior, major and minor axis diameters) and area (TA area and perimeter) parameters as well as tenting volume were predictors for severe fTR. In LSVP group area parameters and leaflet tenting volume lost their predictive significance.

## 4. Discussion

In this prospective study, we analyzed the echocardiographic data of 109 individuals who suffered from fTR due to a variety of different causes (secondary to advanced LSVP and pre-capillary PH). The primary insights gained from our investigation can be summed up as follows: (i) RV diameters, length, areas, and volumes were significantly higher in the pre-capillary PH group compared to LSVP group; (ii) as for 3D TV parameters, PH was associated with larger leaflet tenting height, volume, and more increased indices of septal–lateral and major axis TA diameters; on the contrary, LVSP etiology was associated with higher anterior–posterior TA diameter and sphericity index; and (iii) various etiologies of fTR were associated with different 2D and 3D echocardiographic parameters to predict severe TR.

When left-sided cardiac disease is present, functional TR can develop as a consequence of a raised left atrial pressure that ultimately results in PH. Elevated RV afterload results in a decrease in RV systolic function and associated RV dilation. Significant geometric abnormalities must exist for fTR to occur, but two significant alterations to the normal geometry must be identified: TA dilatation and leaflet tethering [18]. TA dilation has traditionally been described as secondary to RV dilation and dysfunction due to pressure overload, which, in turn, distorts the normal geometry and spatial relationships of leaflets, papillary muscles, and chordae resulting in fTR, which further exacerbates RV and TA remodeling, constituting a vicious circle [19,20].

RV lengthening and eccentricity cause leaflet tethering and tenting with little annular enlargement in patients with fTR and PH [6]. RV dilation, as demonstrated by Spinner et al., results in the displacement of lateral and apical papillary muscles as well as the tethering of leaflets [21]. Using serial echocardiography, it was shown that in PAH, TR progression is associated with both TA dilatation and leaflet tethering, which together contribute to increased tenting area and TV orifice area, which exceeded TV coverage and decreased coaptation area, leading to worsening TR. 

Until now, most research has been focused on the differences of right heart geometry between atrial functional (AF-TR) and ventricular functional TR (VF-TR) [1,22,23,24]. Topilsky et al. analyzed a total of 281 cases with isolated TR (AF-TR) and pulmonary hypertension-related fTR (VF-TR) and reported characteristic TV geometry and RV remodeling [6]. In the former, excess annular and RV-basal enlargement and RV conical deformation did not cause notable valvular tenting, and conversely, in the latter, leaflet tethering with tenting linked to RV elongation and elliptical/spherical deformation correlated with TR severity. Although the different TV geometry characteristic of VF-TR and AF-TR has already been described, the exact pathophysiological mechanism involved in VF-TR due to left valve pathology and VF-TR due to PH remains to be elucidated.

Different fTR etiologies, such as permanent atrial fibrillation, left heart disease (LHD), PH, and corrected tetralogy of Fallot, were found to be associated with varying degrees of TA dilation, leaflet tenting, and right chambers remodeling, according to the findings of Muraru and colleagues [25]. The authors demonstrated that TA was dilated in all fTR patient groups. LHD and particularly PH patients had marked dilation of both RV and RA chambers, as most patients had moderate/severe fTR. Moreover, increased tenting volume was associated with significant RV dilation (PH and LHD). However, the focus of this study was to compare the differences between AF-TR and VF-TR, but not the different etiologies of VF-TR. To date, no study has investigated which right heart geometry and function changes are specific in different etiologies of VF-TR, and which right heart parameters are associated with severe fTR in different etiologies of VF-TR.

Our study analyzed the differences of RV and TV between various etiologies of VF-TR (due to advanced LSVP and pre-capillary PH). Results revealed that not only greater RV dilatation and larger leaflet tethering, but also more impaired RV functional parameters were present in the pre-capillary PH group compared to the LSVP group. Additionally, although TR severity and 2D measured TA diameters did not differ between LSVP and PH groups in our study, 3D echo analysis allowed us to detect subtle TA geometry changes. 

By 3D analysis of the RV and TV geometry using dedicated software, Song et al. showed that the septal–lateral annulus diameter, septal–lateral RV basal diameter, as well as the tenting angles of the septal and anterior leaflets were independent determinants of the severity of TR [18,26]. Nevertheless, our study showed that not only basal RV and septal–lateral TA diameter, but also anterior–posterior TA diameter were independent predictors of TR severity. Additionally, the subgroup analysis of different fTR etiology revealed that TA diameter and area parameters as well as tenting volume were determinants of severe TR in PH group, whereas, in LSVP group, area parameters lost the significance.

When quantifying the right-chambers sizes by 2D echocardiography, significant underestimation may occur due to foreshortening or geometrical assumptions. The 3D echocardiography has revolutionized the non-invasive imaging of the TV apparatus, conferring new insights and better understanding of the pathophysiology of fTR [27]. Our study also demonstrated that 2D echo-derived TA 4-chamber diastolic diameter had a lower predictive value with worse sensitivity and specificity, compared to all 3D TA parameters. The 3D TA analysis provides 82–91% sensitivity for defining severe TR in PH group.

Our study was the first to investigate the predictors of severe TR in different etiologies of fTR. The subgroup analysis demonstrated that different TV parameters (major axis TA diameter and annulus perimeter for PH and septal–lateral TA diameter for LSVP group) had the highest predictive value for severe TR. Among RV parameters, RV middle and basal diameter, and RV end-systolic area were associated with the severe TR in both VF-TR groups.

## 5. Study Limitations

A limitation of our study is that invasive hemodynamic study was not performed in LSVP patients and the pulmonary artery pressures were measured non-invasively in this group of patients. In the precapillary PH group, all patients underwent an invasive hemodynamic study with invasive measurements of mean PAP, systolic PAP, Wedge pressure, and pulmonary vascular resistance. 

## 6. Conclusions

The implementation of 3D echocardiography is useful in the determination of RV and TV geometry changes and might provide valuable insights in the functional TR evaluation. Precapillary PH is related to more severe RV dilatation, dysfunction, and remodeling of TV geometry (larger leaflet tenting volume, more increased indices of septal–lateral and major axis TA diameters, smaller anterior–posterior TA diameter and sphericity index), when compared to the LSVP group. The 2D and 3D echocardiographic parameters help to predict severe TR in different fTR etiologies: major axis TA diameter and annulus perimeter and the leaflet tenting volume have the highest predictive value in PH group, while septal–lateral systolic TA diameter has the highest predictive value in LSVP group of pts. 

## Figures and Tables

**Table 1:** Clinical and 2D echocardiographic characteristics of study population.

	LVSP	PH	Controls	*p*-Value for LSVP vs. PH	*p*-Value for LSVP vs. Controls	*p*-Value for PH vs. Controls
Clinical characteristics						
Age, years	68 (8.55)	58 (14.86)	62 (12.05)	0.001	0.029	0.629
Sex, females %	46.4	72.5	52.6	0.026	0.165	0.029
Body mass index, kg/m^2^	27.9 (5.06)	26.9 (6.14)	27.4 (4.51)	0.374	0.675	0.782
PA pressures						
Max. systolic PAP, mmHg	53.44 (23.25)	73.16 (38.48)	24.16 (5.4)	<0.001	<0.001	<0.001
Mean PAP, mmHg	38 (11.75)	42 (7.5)	14 (9)	0.001	<0.001	<0.001
RA parameters						
RA diameter, mm	50 (9)	56.5 (15.75)	38 (5)	0.004	<0.001	<0.001
RA area, cm^2^	27.8 (10.18)	30.89 (13.17)	17.3 (5.4)	0.484	<0.001	<0.001
RA volume, mL	97.7 (59.26)	130 (70.75)	47.77 (21)	0.286	<0.001	<0.001
RV parameters						
RV parasternal diastolic diameter, mm	36 (7.5)	40 (8.5)	31 (6.5)	0.019	0.003	<0.001
RV basal diameter, mm	45 (7)	52 (11.75)	34 (5)	0.001	<0.001	<0.001
RV middle diameter, mm	36 (9.25)	47 (12.5)	28.5 (3.25)	<0.001	0.001	<0.001
RV length, mm	63 (17.5)	71 (8.72)	66 (11.5)	<0.001	0.504	0.043
RV sphericity index, %	0.57 (0.17)	0.66 (0.19)	0.44 (0.06)	<0.001	<0.001	<0.001
RV end-diastolic area, cm^2^	22 (10.44)	31.14 (15.57)	17.6 (4.98)	<0.001	0.028	<0.001
RV end-systolic area, cm^2^	14 (7.12)	23.1 (14.1)	10 (3.4)	<0.001	0.002	<0.001
FAC, %	0.32 (0.1)	0.25 (0.1)	0.43 (0.08)	<0.001	<0.001	<0.001
TAPSE, mm	17.5 (5.5)	16 (5.25)	24 (7)	0.054	<0.001	<0.001
RV S’, cm/s	10 (5)	9 (2.75)	12 (4.5)	0.039	0.007	<0.001
RV septal wall strain, %	−10.7 (4.75)	−11 (3.9)	−21.1 (3.9)	0.909	<0.001	<0.001
RV lateral wall strain, %	−20.15 (6.3)	−16.6 (6.3)	−25.8 (3.2)	0.028	<0.001	<0.001
TV parameters						
TA diastolic diameter, mm	43 (5)	43 (5.5)	32 (6.25)	0.604	<0.001	<0.001
TA diastolic diameter index, mm/m^2^	21.86 (3.4)	23.22 (5.53)	17.09 (2.95)	0.061	<0.001	<0.001
TA systolic diameter, mm	39 (5.5)	40 (4.75)	29 (6.13)	0.73	<0.001	<0.001
TA systolic diameter index, mm/m^2^	20 (2.54)	21.25 (5.27)	15.62 (2.42)	0.102	<0.001	<0.001
TV leaflet tethering height, mm	5.5 (2.3)	7.8 (3.7)	3.3 (2)	<0.001	<0.001	<0.001
TV tenting area (cm^2^)	1.25 (0.7)	1.79 (1.04)	0.68 (0.32)	<0.001	<0.001	<0.001
TV EROA, mm^2^	28.94 (20.97)	29.96 (23.76)		0.464		

EF—ejection fraction, RV—right ventricle, FAC—fractional area change, TAPSE—tricuspid annular plane systolic excursion, RV S’—tricuspid lateral annular systolic velocity, RA—right atrium, PA—pulmonary artery, TA—tricuspid annulus, TV—tricuspid valve, EROA—effective regurgitant orifice area, LSVP—left-sided valvular pathology, PH—pulmonary hypertension.

**Table 2:** The 3D echocardiographic characteristics of study population.

	LVSP	PH	Controls	*p*-Value for LSVP vs. PH	*p*-Value for LSVP vs. Controls	*p*-Value for PH vs. Controls
RV parameters						
RV end-diastolic volume, mL	147.05 (103.08)	182.75 (105.03)	85.5 (86.75)	0.007	0.003	<0.001
RV end-systolic volume, mL	86.65 (65.23)	129.5 (76.68)	44.9 (46.9)	0.003	<0.001	<0.001
RV EF, %	40.5 (6.73)	30.85 (10.48)	49.8 (5.85)	0.001	<0.001	<0.001
TV parameters						
TA area, cm^2^	14.3 (4.4)	13.2 (3.4)	8.1 (0.93)	0.649	<0.001	<0.001
TA area index (cm^2^/m^2^)	7.13 (2.25)	6.88 (2.17)	4.45 (0.97)	0.713	<0.001	<0.001
TA perimeter, mm	130 (23)	133 (17)	102 (7.5)	0.979	<0.001	<0.001
TA perimeter index (cm/m^2^)	66.4 (15.8)	71.1 (19.3)	55 (9.8)	0.065	0.004	<0.001
Septal-Lateral Systolic TA Diameter, mm	42 (5)	44 (7)	33.5 (4.5)	0.37	<0.001	<0.001
Septal-Lateral Systolic TA Diameter Index, cm/m^2^	21.5 (3.1)	23.6 (7.1)	18.1 (2.7)	0.021	0.003	<0.001
Septal-Lateral Diastolic TA Diameter, mm	45 (5)	46 (7)	36.5 (5.8)	0.329	<0.001	<0.001
Septal-Lateral Diastolic TA Diameter Index, mm/m^2^	22.9 (4)	25.1 (4.9)	19.1 (1.8)	0.023	0.006	<0.001
Anterior–Posterior TA Diameter, mm	40 (8)	35 (7)	28 (4.5)	0.037	<0.001	<0.001
Anterior–Posterior TA Diameter Index, mm/m^2^	20.3 (4.7)	18.8 (5.5)	15.6 (4.5)	0.411	<0.001	0.003
Major Axis Systolic TA Diameter, mm	46 (5)	46 (6)	36 (2.8)	0.791	<0.001	<0.001
Major Axis Systolic TA Diameter Index, mm/m^2^	23.2 (4.7)	25 (5.2)	20.1 (1.9)	0.074	0.003	<0.001
Major Axis Diastolic TA Diameter, mm	48 (6.5)	48 (8)	39.5 (5)	0.777	<0.001	<0.001
Major Axis Diastolic TA Diameter Index, mm/m^2^	23.9 (4.4)	26.2 (5.2)	20.3 (2.8)	0.044	0.006	<0.001
Minor Axis Diastolic TA Diameter, mm	39 (8)	36 (6)	27 (3.5)	0.222	<0.001	<0.001
Minor Axis Diastolic TA Diameter Index, mm/m^2^	19.8 (4.4)	19.7 (5.9)	15.2 (3.7)	0.908	<0.001	<0.001
TV Leaflet Coaptation point Height, mm	9 (5.5)	13 (3)	6.5 (2)	<0.001	0.012	<0.001
TV Leaflet Tenting Volume, mL	3.9 (2)	5 (2.9)	1.55 (0.25)	0.025	<0.001	<0.001
TV Sphericity Index, %	83.67 (11.33)	80 (14.04)	73.61 (12.4)	0.04	0.002	0.13

RV—right ventricle, EF—ejection fraction, TV—tricuspid valve, TA—tricuspid annulus, LSVP—left-sided valvular pathology, PH—pulmonary hypertension.

**Table 3:** Relation of RV and TV geometry parameters with severe fTR.

	Univariate			Multivariate		
	OR	95% CI	*p*-Value	OR	95% CI	*p*-Value
3D echo-derived TV parameters						
TA area, cm^2^	1.434	1.163–1.769	<0.001			
TA perimeter, mm	1.058	1.021–1.096	0.002	0.421	0.221–0.975	0.042
Septal-Lateral Systolic TA Diameter, mm	1.507	1.233–1.842	<0.001	1.651	1.042–2.385	0.028
Septal-Lateral Diastolic TA Diameter, mm	1.431	1.193–1.718	<0.001			
Major Axis Systolic TA Diameter, mm	1.386	1.176–1.632	<0.001			
Major Axis Diastolic TA Diameter, mm	1.363	1.165–1.594	<0.001			
TV Leaflet Tenting Volume, mL	1.580	1.177–2.120	0.002			
2D echo-derived TV parameter						
4-Chambers Systolic Diameter, mm	1.255	1.106–1.424	<0.001			
RV parameters						
2D RV basal diameter, mm	1.149	1.073–1.230	<0.001	1.198	1.021–1.409	0.029
2D RV middle diameter, mm	1.083	1.036–1.133	<0.001			
RV end-diastolic area, cm^2^	1.096	1.042–1.152	<0.001			
RV end-systolic area, cm^2^	1.112	1.050–1.178	<0.001			
RV EF, %	0.911	0.855-.0971	0.004			

TA—tricuspid annulus, TV—tricuspid valve, RV—right ventricle, EF—ejection fraction.

**Table 4:** Relation of 3D TV geometry and severity of fTR in different TR etiologies.

	LSVP			PH		
	OR	95% CI	*p*-Value	OR	95% CI	*p*-Value
TA area, cm^2^	1.282	1.001–1.641	0.05	2.198	1.138–4.244	0.02
TA perimeter, mm	1.032	0.995–1.070	0.09	1.242	1.033–1.495	0.02
Septal-Lateral Systolic TA Diameter, mm	1.430	1.123–1.820	<0.001	1.717	1.149–2.566	0.01
Septal-Lateral Diastolic TA Diameter, mm	1.394	1.108–1.754	<0.001	1.498	1.089–2.062	0.01
Anterior-Posterior TA Diameter, mm	1.147	0.999–1.316	0.051	1.246	1.022–1.518	0.03
Major Axis Systolic TA Diameter, mm	1.268	1.066–1.508	0.01	1.934	1.149–3.256	0.01
Major Axis Diastolic TA Diameter, mm	1.253	1.059–1.484	0.01	1.716	1.154–2.550	0.01
Minor Axis Diastolic TA Diameter, mm	1.118	0.976–1.281	0.11	1.228	1.009–1.493	0.04
TV Leaflet Tenting Volume, mL	1.398	0.995–1.965	0.53	2.149	1.143–4.040	0.02

TA—tricuspid annulus, TV—tricuspid valve, LSVP—left-sided valvular pathology, PH—pulmonary hypertension.

## Data Availability

The data presented in this study are available on request from the corresponding author. The data are not publicly available due to privacy reasons.

## Supplementary Materials

The following supporting information can be downloaded at: https://www.mdpi.com/article/10.3390/medicina59010057/s1, Table S1: ROC analysis of severe fTR for all cohort.; Table S2: Prediction of severe fTR for patients with different aetiologies.
