# Supplementary material for: Different Causes of Functional Tricuspid Valve Regurgitation Are Linked to Differences in Tricuspid Valve and Right-Sided Heart Geometry and Function: 3D Echocardiography Study
Source: Medicina (Kaunas). 2022 Dec 27;59(1):57. doi: 10.3390/medicina59010057 (PMC9860866; doi:10.3390/medicina59010057)
Supplement: Supplementary file 1 [file medicina-59-00057-s001.zip › medicina-1980453-supplementary.pdf]

Supplement Table S1. ROC analysis of severe fTR for all cohort.

Variables	AUC	95% CI	p-value	J index
**2D RV parameters**				
RV basal diameter, mm	**.767**	.675-.860	<.001	.437
RV middle diameter, mm	**.744**	.651-.837	<.001	.392
RV length, mm	.664	.554-.773	.006	.258
RV sphericity index, %	.645	.527-.763	.016	.295
RV end-diastolic area, cm2	**.724**	.621-.827	<.001	.407
RV end-systolic area, cm2	**.729**	.629-.828	<.001	.370
FAC, %	.687	.579-.794	.002	.375
TAPSE, mm	.683	.574-.791	.003	.304
RV S', cm/s	.629	.519-.739	.032	.231
RV septal wall strain, %	.550	.276-.824	.734	.200
RV lateral wall strain, %	.450	.176-.724	.734	.100
**2D TV parameters**				
TA diastolic diameter, mm	**.725**	.529-.821	<.001	.356
TA diastolic diameter index, mm/m2	.697	.590-.805	.001	.362
TA systolic diameter, mm	.688	.587-.789	.002	.272
TA systolic diameter index, mm/m2	.673	.562-.783	.004	.301
**3D RV parameters**				
RV end-diastolic volume, ml	.645	.525-.764	.034	.288
RV end-systolic volume, ml	.666	.547-.785	.015	.299
RV EF, %	**.700**	.581-.818	.003	.379
**3D TA parameters**				
TA area, cm2	**.738**	.623-.854	<.001	.412
TA area index (cm2/m2)	.688	.563-.812	.006	.379
TA perimeter, mm	**.735**	.616-.854	.001	.396
TA perimeter index (cm/m2)	.674	.552-.797	.011	.275
Septal-Lateral Systolic TA Diameter, mm	**.826**	.734-.917	<.001	.456
Septal-Lateral Systolic TA Diameter Index, cm/m^2	**.724**	.610-.837	.001	.390
Septal-Lateral Diastolic TA Diameter, mm	**.805**	.706-.904	<.001	.440
Septal-Lateral Diastolic TA Diameter Index, mm/m^2	.677	.556-.799	.009	.352
Anterior – Posterior TA Diameter, mm	.691	.569-.813	.005	.385
Anterior – Posterior TA Diameter Index, mm/m2	.643	.521-.766	.035	.335
Major Axis Systolic TA Diameter, mm	**.816**	.718-.913	<.001	.533
Major Axis Systolic TA Diameter Index, mm/m2	.679	.557-.801	.008	.299

Major Axis Diastolic TA Diameter, mm	**.812**	.710-.914	<.001	.574
Major Axis Diastolic TA Diameter Index, mm/m^2	.682	.561-.804	.007	.283
Minor Axis Diastolic TA Diameter, mm	.657	.525-.788	.021	.275
Minor Axis Diastolic TA Diameter Index, mm/m^2	.609	.471-.747	.111	.242
TV Leaflet Coaptation point Height, mm	.577	.443-.712	.256	.132
TV Leaflet Tenting Volume, ml	**.708**	.583-.833	.002	.385
TV Sphericity Index, %	.623	.499-.746	.072	.294

RV – right ventricle, EF – ejection fraction, TV – tricuspid valve, TA – tricuspid annulus.

Supplement Table S2. Prediction of severe fTR for patients with different aetiologies.

	LVSP					PH				
Variables	AUC	p-value	Cut-off	Sensitivity	Specificity	AUC	p-value	Cut-off	Sensitivity	Specificity
**3D TV parameters**										
TA perimeter, mm	0.653	0.07				0.906	**<0.001**	134	82	81
TA area, cm²	0.678	**0.04**	14	65	61	0.886	**<0.001**	13	82	81
TV Leaflet Tenting Volume, ml	0.627	0.14				0.824	**<0.001**	48	82	63
Septal-Lateral Systolic TA Diameter, mm	0.761	**<0.001**	43	71	61	0.898	**<0.001**	45	82	81
Anterior-Posterior TA Diameter, mm	0.676	**0.04**	40	65	56	0.741	**0.04**	36	64	63
Major Axis Systolic TA Diameter, mm	0.747	**<0.001**	47	76	67	0.929	**<0.001**	47	91	81
**2D TV parameters**										
4-Chambers Systolic Diameter, mm	0.706	**0.008**	43	70	55	0.741	**0.013**	44	71	64
4-Chambers Diastolic Diameter, mm	0.656	**0.043**	39	75	45	0.719	**0.025**	41	64	64
**RV parameters**										
RV basal diameter, mm	0.765	**<0.001**	46	75	67	0.774	**0.005**	52	79	60
RV middle diameter, mm	0.768	**<0.001**	36	85	63	0.790	**0.003**	48	71	64
RV length, mm	0.709	**0.01**	64	65	61	0.546	0.639			
RV end-systolic area, cm2	0.764	**<0.001**	15	80	67	0.700	**0.040**	23	79	60
RV EF, %	0.636	0.114				0.786	**0.012**	29	76	73

TA - tricuspid annulus, TV - tricuspid valve, RV - right ventricle, EF - ejection fraction, LSVP – left-sided valvular pathology, PH – pulmonary hypertension
